# Exploring Self-regulation of More or Less Expert College-Age Video Game Players: A Sequential Explanatory Design

**DOI:** 10.3389/fpsyg.2016.01441

**Published:** 2016-09-27

**Authors:** Meryem Yilmaz Soylu, Roger H. Bruning

**Affiliations:** ^1^Formerly affiliated with Meliksah UniversityKayseri, Turkey; ^2^Department of Educational Psychology, University of Nebraska-LincolnLincoln, NE, USA

**Keywords:** self-regulation, video game, expertise, mixed-methods, a sequential explanatory design

## Abstract

This study examined differences in self-regulation among college-age expert, moderately expert, and non-expert video game players in playing video games for fun. Winne's model of self-regulation (Winne, 2001) guided the study. The main assumption of this study was that expert video game players used more processes of self-regulation than the less-expert players. We surveyed 143 college students about their game playing frequency, habits, and use of self-regulation. Data analysis indicated that while playing recreational video games, expert gamers self-regulated more than moderately expert and non-expert players and moderately expert players used more processes of self-regulation than non-experts. Semi-structured interviews also were conducted with selected participants at each of the expertise levels. Qualitative follow-up analyses revealed five themes: (1) characteristics of expert video gamers, (2) conditions for playing a video game, (3) figuring out a game, (4) how gamers act and, (5) game context. Overall, findings indicated that playing a video game is a highly self-regulated activity and that becoming an expert video game player mobilizes multiple sets of self-regulation related skills and processes. These findings are seen as promising for educators desiring to encourage student self-regulation, because they indicate the possibility of supporting students via recreational video games by recognizing that their play includes processes of self-regulation.

## Introduction

The best-selling game of 2014 [ESA (Entertainment Software Association), 2015], *Call of Duty: Advanced Warfare*, has been played over a billion hours since its release in November 2014, while the *PEW Teens, Social Media, Technology Overview* (Lenhart et al., 2015) has reported that 42% of Americans play games regularly for 3 h or more in a week and 81% of American youth now can access a game console. This means that the average young individual in the US ages 12–17 may spend hundreds of hours annually playing video games and will have had thousands of hours of experience with video games in a 10-year span. An advanced player, of course, may spend many more hours than this (VanDeventer and White, 2002; Green and Bavelier, 2003; West et al., 2008; Hubert-Wallander et al., 2011). Do we consider these hours wasted, or do video game players (video gamers) learn things while playing a video game that have more general cognitive function? Perhaps the players are acquiring something that is highly valued in many domains—expertise (in this case expertise in playing video games).

Experts are recognized for their extensive experience and deep knowledge in a given field, but we also know that becoming an expert is not an easy process. It is generally accepted that one typically needs to study and practice thousands of hours in a domain in order to become an expert (Ericsson et al., 1993; Chi, 2006). But it is also recognized that acquiring expertise is worth the effort, because experts possess a rich repertoire of tactics and sophisticated methods for solving specific kinds of problems, which allows them to solve such problems faster and more effectively than non-experts (Chi et al., 1988; Ericsson et al., 1993; Chi, 2006). Moreover, experts work with organized knowledge and spend more time analyzing the problem at hand. They typically use pattern recognition, often reaching solutions with few or no errors (Chi et al., 1988; Stubbart and Ramaprasad, 1990; Wenning, 2002).

A number of researchers (e.g., Winne, 1997; Schunk and Zimmerman, 1998; Boekaerts et al., 2000; Zimmerman and Schunk, 2001; Zimmerman, 2006) have shown that many characteristics present in experts' repertoires (e.g., using task analysis, goal setting, strategy choice) are also essential components of self-regulation (SR). Experts report using many different kinds of SR processes including planning, setting goals, self-observation, and self-evaluation (Zimmerman, 2002a).

In an early study of whether expertise can be demonstrated in the context of video games, VanDeventer and White (2002) found that outstanding video game players did in fact show many of the attributes of experts, including actively seeking new information, integrating new with prior information, assessing current states, utilizing a variety of kinds of data, organizing and categorizing inputs, consciously repeating successful behavior, making corrections as needed, recognizing patterns, constraints, and misinformation, and using inductive, critical, and holistic thinking. In addition, video game experts were more efficacious about their abilities, likely to take risks, able to analyze a variety of inputs, and capable of integrating knowledge with behavior.

Beyond interacting with the specific content of a video game, video game players also may be learning more generally to regulate their cognitive, affective, and behavioral processes as they cope with the wide range of information and decisions many games require. As players engage with games, they must make inferences, judgments and decisions about their own actions and their consequences and use these to adjust and adapt their goals. For instance, if video gamers are cognizant that they have to reach a certain score to play the next level of the game, they will tend to self-regulate their play to successfully complete the current level (Zaparyniuk, 2006).

Although much has been written generally about how experts and non-experts self-regulate in various domains and whether they can be distinguished in terms of levels of self-regulation (Lefebvre-Pinard and Pinard, 1985; Glaser, 1987; Zimmerman and Risemberg, 1997; Zimmerman, 1998, 2002a,b; Cleary and Zimmerman, 2001), information on how video game playing college students in different expertise groups self-regulate their video game play has been lacking. The current study therefore was aimed at better understanding college students' self-regulation experiences in recreational video gaming. As shown in prior research in a variety of other contexts, experts and non-experts have been shown to differ in use of self-regulation (e.g., Zimmerman and Risemberg, 1997). This study's goal was to determine whether college age video game players of different expertise levels likewise could be distinguished by their use of self-regulation in recreational video game playing, specifically whether more expert game players use more processes of self-regulation than less expert players as they played a video game for fun. The following quantitative and qualitative research questions (RQs) guided the study.

Quan RQ: Do video game playing college students in different expertise groups differ in their use of self-regulation as playing video game for fun?

Qual RQ: Do video game playing students in different expertise groups explain their usage of self-regulation differently?

In order to bring quantitative and qualitative parts of the research together, a mixed methods question then was utilized to guide the study, framed within a sequential explanatory design (QUAN 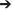 qual).

Mixed Methods RQ: To what extent do themes emerging from participants' statements inform interpretation of quantitative differences observed among different expertise groups in use of self-regulation in recreational video game play of college-age video game players?

## Theoretical framework

A variety of definitions exist for self-regulated learning (SRL), each tracing back to one or more different self-regulated learning models that offer different perspectives on how self-regulated learners function (Winne and Hadwin, 1998; Zimmerman, 1998; Pintrich, 2000; Hadwin and Oshige, 2011). Some models emphasize the social nature of self-regulation (Zimmerman, 1998; Pintrich, 2000) while others focus more on information processing (Pressley et al., 1989; Winne and Hadwin, 1998), the cognitive operations necessary for self-regulating (Boekaerts, 1997), the situated and dynamic nature of SRL (Hadwin, 2000; Hadwin and Oshige, 2011) or cognition-behavior relationships in self-regulation (Carver and Scheier, 1981; Baumeister and Vohs, 2007).

Video game and learning environments more generally arguably share considerable common ground (Gee, 2003, 2007; Gee and Hayes, 2009). Among fundamental principles appearing to underlie both video games and effective learning contexts are their being goal oriented, requiring use of certain strategies, and providing feedback on progress (Rigby and Przybylski, 2009). According to Winne's (2001) Four-Phase Self-Regulated Learning (SRL) model, which provided the theoretical base for this study, when more highly self-regulated learners face a learning task, they consider their prior knowledge, beliefs, environmental structuring, time, and repertoire of study tactics and try to understand what a task asks of them. They then set goals and plan how to learn, apply, and adapt study tactics to achieve their learning goals.

The Winne model includes four phases—task definition, goal setting and planning, enactment, and adaptation. In Phase 1, *task definition*, learners generate perceptions of the task at hand based on previous experiences and current task conditions. Phase 2, *goal setting and planning*, is devoted to forming goals and planning, while Phase 3, *enactment*, involves enacting tactics and strategies planned in the goal setting and planning phase. Phase 4, the final phase, requires *adapting tactics and strategies* by which learners monitor their progress and make changes as necessary for future needs.

When playing a video game, although it may seem as if conditions in the *task definition* phase and standards in the *goal setting and planning* phase are directed by the video game developers, the opposite is also true. Player qualifications, decisions, and perspectives from players' own social environments most likely shape game play because their competences such as tactic repositories as well as the perception of assistance coming from the social environment may actually help them understand how to maximize their success. In the goal setting and planning phase, players may form their own personal goals for play based upon the outcomes of the task definition phase.

When we think about the *enactment* phase in video game playing, players may react to inputs (e.g., new weapon or extra score) in order to win a battle or progress in the game. Inputs may include the information presented as products of the task definition (e.g., getting together with a friend to play) and goal setting and planning phases (e.g., beating an opponent). In the enacting tactics phase, players may create new products using new approaches (e.g., new moves to get more coins) and evaluating the effectiveness of these tactics in comparison to standards. Finally, in the *evaluation and adaptation phase*, video game players may assess their whole game play; then, based on their analysis, they may decide to abandon the way they previously played or search for new ways to play in the game's social world, in other words in the game's affinity spaces (Gee and Hayes, 2009).

Winne's theoretical model of self-regulation was selected to frame the present study for several reasons. First, this model includes the possibility that learners—in this case video game players—may be seeking more than one goal simultaneously. For instance, a video gamer may be aiming to win a game with the highest score while also exploring game tricks to share with others. Second, the model provides an explanation for how internal/external feedback about the gap between current and targeted states is utilized by learners to update goals and contextual inputs. For example, screen tips such as score level, remaining lives, and so on can provide information about players' current states so that they can strategize their play for winning. Third, Winne's model portrays SRL as recursive, with each SRL phase influencing other phases both forward and backward, characteristics matching well with the nature of video gaming activity. In both Winne's model generally and in game contexts specifically, phases of activity do not necessarily follow each other sequentially. That is, in certain contexts and times learners/players may skip some phases and revisit them only as needed. Finally, the Winne SRL model is iterative, which means the outcome or products of one or all phases can feed back into other phases as well as into the contextual and social environment (Winne and Hadwin, 1998; Hadwin, 2000; Winne, 2001), characteristics clearly present in many if not most game contexts.

## Methods

This research utilized a mixed methods approach, which is especially well suited for studying complex characteristics of environments in which researchers typically are interested in both user behaviors and contexts (e.g., Solomon, 1997; Fabritius, 1999). The specific framework chosen for the study was a sequential explanatory mixed methods (QUAN
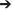
qual) design (Creswell and Plano Clark, 2007), in which qualitative data are collected after a quantitative phase in order to explain the quantitative data in more depth. In the first phase, quantitative data were collected using the Video Game Playing Survey (VGPS), which was designed to provide information about whether college age players of recreational video game in different expertise groups could be distinguished by the self-regulatory processes they reported using while playing these games. Information from this first phase then was further explored in a second qualitative phase involving one-on-one interviewing. In this explanatory follow-up, interview questions were constructed based on the quantitative results (Creswell and Plano Clark, 2007) and semi-structured interviews conducted with a sample of students from each expertise group. The interviews focused on gaining more detailed understanding about when and how video gaming expertise might be tied to players' self-regulation during video game play.

## Phase 1: quantitative data collection, analysis, and results

### Quantitative participants and data collection

Using convenience sampling, college students 19 years of age and older were recruited from educational psychology courses at a large Midwestern university. These students were targeted through courses required for all students seeking teaching-related degrees in the college of education and human sciences at the university, so the sample was likely to represent this population. Although it was recognized that using probability samples would aid in generalizing the results of this study to all college students (Rosenfeld, 2012), convenience sampling was utilized due to limitations on our access to the participant group. These limitations are explicitly accounted for in our data analysis and interpretations, however.

In the first, quantitative phase of this study, a cross sectional survey design was deployed in which information was gathered from all participants. One hundred forty-three college students enrolled in undergraduate educational psychology courses at a large Midwestern university agreed to take the survey. Fourteen did not complete the online VGPS, leaving a total of 129 participants. Of these participants, 73% (94) were female and 27% (35) were male. Student reporting their ethnicity as Caucasian comprised 88% of the sample, while 3% were African-American, 2% were Asian/Pacific Islander, and 7% were multiracial and other. Thirty-two (24.8%) students identified themselves as sophomores, 52 (40.3%) indicated they were juniors, and 43 (33.3%) reported they were seniors. Two participants (1.6%) were graduate students.

Of this group, 87 participants (67.4%) labeled themselves as non-expert video gamers, five (3.9%) reported that they considered themselves expert video gamers, and 37 (28.7%) indicated that they were moderately expert video gamers. Participants also were asked to estimate the number of years they had been playing video games. This number ranged from zero to 26 years, with a mean of 9.8 years, a median of 10 years, and a mode of 15 years.

All needed permissions were obtained from the course supervisor, course instructors, and the university's Institutional Review Board (IRB) process prior to presenting the survey to students in the targeted courses. Those students who agreed to participate were administered the VGPS, which included three sections:

1. *General information about participants' video game playing*. This section of the survey inquired about general video gaming habits and expertise level and included eight items asking participants about the hours they played video games and experiences with games of various genres. In order to permit identification of participant subgroups based on their expertise in video game playing, questions (see Table 1) focused on amount of time they played video games in the past week, in a week in which classes are in session, and during a week when there were no classes.
2. *Playing my video game scale (PMVGS).* The second part of the survey consisted of a measure, the Playing My Video Game Scale (PMVGS), in which participants were questioned about their actual processes of self-regulation during recreational gaming. The PMVGS scale was constructed to reflect each of the four posited categories of Winne's (2001) model of self-regulated learning to permit inferences about the extent to which each was utilized in the gaming context. Of the PMVGS's 42 items 10 were focused on task definition, six on goal setting and planning, 14 on enacting tactics, and 12 on adapting tactics. Participants were asked to rate how often they did each of the activities included in the scale by indicating a percentage from 0 (never) to 100 (always).

**Table 1:** **Sample items from first section of the VGPS**.

**1. In the past week**, about how many **total hours** have you spent playing video games? ____hours/week				
**2. During a typical week when classes are in session**, about how many total hours do you spend playing video games? ____hours/week				
**3. When there is no class**, about how many total hours do you spend playing video games? ____hours/week				
4. Please list **5 video games** that you play most often. For each, report how many hours in a typical week you play each of them. (if you don't play as many as 5 video games, list only those you do play and numbers of hours you play each of them)				
a. __________________________________________ ____hours/week				
Think about your video gaming expertise by responding to each of the following. For each item, rate your expertise level by indicating a percentage from 0 (not expert at all) to 100 (highly expert). The scale below is for reference only; you don't need to use only the given values. You may assign **any number** between 0 and 100 (e.g., 26.87) as your rating.				
0	25	50	75	100
Not expert at all	Not very expert	Somewhat expert	Expert	Highly expert
a. _____ Your playing video games **in general**				
b. _____ The video game you play **most often**				

Preliminary versions of the PMVGS were further refined through discussion with faculty members and graduate students with expertise in video games and educational research. It then was pilot-tested with a set of college students (*N* = 20) from the same general participant pool as the study's primary participants. Cronbach's alpha reliability for the overall scale in the pilot study was 0.94 but due to the limited number of participants in the pilot study, however, exploratory factor analysis (EFA) was not conducted to find possible subscales of the PMVGS. Based on results from the pilot test, a near-final set of items once again was reviewed by the researchers for final edits in wording and methods of scale administration made to make the measure more suitable for college age video game playing students.

In the study itself, however, EFA was used to determine if there were possible subscales or results at any level corresponding to the dimensions proposed by Winne and colleagues (Winne and Hadwin, 1998; Winne, 2001). Based on this analysis, it was concluded that a single primary component including all 26 items (see Table 2) best represented game-related self-regulation processes. Once the unifactor structure of the *PMVGS* was determined, an internal consistency estimate (Cronbach's alpha) was calculated, which again showed the scale to have high reliability (α = 0.97).

3. *Information on participant demographics*. This part of the survey provided information regarding participants' gender, ethnicity, and academic standing in order to describe participants. Participants' names and email addresses also were collected to permit further contact with them if they had agreed to participate in and were selected for the qualitative part of this research.

**Table 2:** **Exploratory factor analysis results for PMVGS scores using principal factors extraction**.

**Item no**	**Item**	**Loading for the 1st factor**
1.	I review my movements to see my progress.	0.823
2.	I communicate with other gamers to find out how well I am playing.	0.821
3.	I find myself analyzing the usefulness of strategies while I play.	0.811
4.	I frequently evaluate my progress.	0.807
5.	I find myself pausing regularly to check my play.	0.807
6.	I plan the things I have to do next in order to complete the game.	0.804
7.	I use game help menus to find solutions to game challenges.	0.803
8.	I think of new ways to pass a challenging level of a game.	0.793
9.	I know the physical places where I can play the best.	0.785
10.	I watch other people play and then I do what they do to beat the game.	0.774
11.	I play a game over and over again to beat the game.	0.773
12.	I set specific goals for myself (e.g., Today I will beat level 60).	0.767
13.	I ask myself if I have considered all options, when playing challenging levels of a game.	0.764
14.	I use my own strategies to get through each level.	0.763
15.	I monitor my playing to pass a challenging level of a game.	0.759
16.	I ask myself if there was an easier way to complete a game after I finish one.	0.759
17.	I play with a partner to progress more quickly.	0.755
18.	I explore and try different things until I find something to beat the game.	0.751
19.	I play as long as I can.	0.750
20.	I consult with my gamer friends to get help about a game.	0.750
21.	I check my score to see my progress in a game.	0.744
22.	I would stay up all night if it meant a high score in a game.	0.743
23.	I know which level I will reach before I stop playing.	0.740
24.	I am open to suggestions to improve my play.	0.673
25.	I would rather play a game than spend time with friends and family.	0.621
26.	I pretend I am one of the best players in this game and play like them.	0.545

Ten research sessions during which participants could complete the online survey were scheduled over a period of 5 days in a campus computer lab. The survey was administered in the computer lab in order to track participating students for purposes of awarding research credits in their classes and encouraging participants to take the survey seriously and answer honestly, while simultaneously maintaining their confidentiality. During these sessions, volunteer session proctors provided general instructions and guidance, and recorded their class section numbers and instructors.

The first page of the online survey included an informed consent form. Students who wished to participate in the study clicked an *agree* button to indicate their consent. In general, participants took approximately 15–20 min to complete the survey, but were able to disengage from taking the survey at any time.

### Quantitative data analysis and results

Quantitative data analysis proceeded in two steps, beginning with initial analyses that were utilized to divide participants into expertise groups. In this first step, two cluster analysis methods, hierarchical and k-means clustering (Punj and Stewart, 1983; Wang et al., 2002; Hair et al., 2006), were performed utilizing items 1, 2, 3, 4a, 5a, and 5b from the first part of the VGPS (see Table 1) to divide the participants into expertise groups. Questions 1, 2, 3, and 4a addressed the amount of time spent playing video games (in the past week, in a typical week when classes were in session, and in a typical week when there were no classes, plus the amount of hours spent playing the game that they played most often). Participants' self-reported video gaming expertise level in general and in their most played video game (both rated on a 100-point scale) were queried in questions 5a and 5b respectively.

Hierarchical cluster analysis showed the presence of three main clusters in the participant group. In order to validate these three clusters, data were analyzed via non-hierarchical k-means cluster analysis in which a three-cluster solution was used as an input (Hair et al., 2006). The three-cluster solution again appeared to be of adequate interpretability but, given the subjective nature of cluster analysis (Salvador and Chan, 2004), the stability of the three cluster solution was further assessed by splitting the data set in half (*n* = 65) and re-running the analysis, thus providing further evidence of the validity and practical significance of the three cluster solution. When these results were compared with the participants' original cluster assignments, 77% of the subjects were classified correctly allowing for confidence in distinct and stable clusters according to participants' amount of hours spent playing video games and video gaming expertise.

The three clusters were utilized to represent overall video gaming expertise levels (see Table 3), with Cluster 1 (*n* = 64) representing participants who were not video game experts, Cluster 2 (*n* = 55) representing participants who were moderately expert, and Cluster 3 (*n* = 10) representing expert video game players.

**Table 3:** **Means and standard deviations for the clustering variables for the three cluster groups**.

**VGPS Items**	**Cluster 1 Not an expert (*n* = 64)**		**Cluster 2 Moderately expert (*n* = 55)**		**Cluster 3 Expert (*n* = 10)**	
	***M***	***SD***	***M***	***SD***	***M***	***SD***
1. Total hours spent with video gaming in the past week (hour per week)	0.59	0.84	2.51	2.99	16.30	8.38
2. Total hours in a typical week spent with video gaming when classes are in session (hour per week)	0.58	0.81	2.13	2.32	14.80	4.52
3. Total hours in a typical week spent with video gaming when there is no class (hour per week)	1.01	1.21	5.02	5.05	23.30	5.58
4a. Total hours in a typical week spent playing the game that they play most often (hour per week)	0.67	0.74	1.98	1.96	10.40	3.81
5a. Self-reported video gaming expertise in general (100pt scale)	12.60	12.27	54.15	23.57	74.42	15.10
5b. Self-reported video gaming expertise in the game they play most often (100pt scale)	26.31	20.97	81.78	13.06	86.93	13.35

An ANOVA was performed in order to identify any significant differences between the clusters (See Tables 4, 5). Significant differences were found between the clusters for all items (four items asking the amount of hours spending playing video game in a week and two items asking level of video gaming expertise). Follow-up *post-hoc* analyses showed that differences between the clusters were in accordance with the cluster interpretation.

**Table 4:** **Comparison of total hours of video gaming per week by within and between three expertise groups**.

**Items in first part of VGPS**		***SS***	***df***	***MS***	***F***	***p***
1. Total hours spent with video gaming in the past week (hours per week)	Between groups	2134.86	2	1067.43	115.82	0.001
	Within groups	1161.28	126	9.22		
	Total	3296.14	128			
2. Total hours in a typical week spent with video gaming when classes are in session (hour per week)	Between groups	1753.67	2	876.84	213.82	0.001
	Within groups	516.70	126	4.10		
	Total	2270.37	128			
3. Total hours in a typical week spent with video gaming when there is no class (hours per week)	Between groups	4327.23	2	2163.62	155.66	0.001
	Within groups	1751.31	126	13.90		
	Total	6078.54	128			
4a. Total hours in a typical week spent playing the game that they play most often (hours per week)	Between groups	818.08	2	409.04	138.45	0.001
	Within groups	372.27	126	2.96		
	Total	1190.36	128			

p < 0.001 represents significant differences within and between the three expertise groups.

**Table 5:** **Comparison of self-reported video gaming expertise by within and between three expertise groups**.

**Items in first part of VGPS**		***SS***	***df***	***MS***	***F***	***p***
5a. Self-reported video gaming expertise in general (100 pt scale)	Between groups	67819.73	2	33909.86	102.83	0.001
	Within groups	41549.12	126	329.76		
	Total	109368.84	128			
5b. Self-reported video gaming expertise in the game they play most often (100 pt scale)	Between groups	102277.65	2	51138.83	167.30	0.001
	Within groups	38515.75	126	305.68		
	Total	140793.40	128			

As seen in Table 4, responses from expert, moderately expert, and non-expert groups showed a significant difference in the number of hours spent playing video game in the past week [*F*_(2, 126)_ = 115.82, *p* < 0.001], in a typical week when classes were in session [*F*_(2, 126)_ = 213.82, *p* < 0.001], in a typical week when there were no classes [*F*_(2, 126)_ = 155.66, *p* < 0.001], and in a typical recent week in which they played their most played game [*F*_(2, 126)_ = 138.45, *p* < 0.001].

Table 5 shows that expert, moderately expert, and non-expert groups significantly differed in video gaming expertise in general [*F*_(2, 126)_ = 102.83, *p* < 0.001] and in the game they played most often [*F*_(2, 126)_ = 167.30, *p* < 0.001]. In conclusion, it could be said that these groups clearly were distinct from one another.

In order to examine if there were any differences among these groups in terms of self-regulation of video gaming, the researchers conducted one-way analysis of variance utilizing participants' self-regulation of gaming scores, which was the sum of *PMVGS* item scores. This analysis revealed significant differences [*F*_(2, 126)_ = 25.79, *p* < 0.001] among expert, moderately expert, and non-expert video game players. *Post-hoc* follow up analyses using the Scheffe criterion for significance (Day and Quinn, 1989) indicated that expert video gamers (*M* = 1238.68, *SD* = 488.41) self-regulated more than moderately expert video gamers (*M* = 633.75, *SD* = 543.59), and that non-expert video gamers (*M* = 251.70, *SD* = 339.07), and moderately expert gamers self-regulated more than non-experts (*M* = 251.70, *SD* = 339.07).

It should be noted that the scores of experts and non-experts were significantly different (*p* < 0.01) on all of the self-regulation items, indicating that experts reported utilizing a wider variety of self-regulation processes during their video game playing compared to non-experts. For the majority of items, similar differences appeared in comparisons of expert and moderately expert players.

Considering Winne's model, significant differences between the expert and moderately expert players on the first three items (Items 1, 2, 3, see Table 6) are consistent with the claim that expert and moderately expert players have quite different ideas about the conditions under which they play video games. Similarly, responses from expert and moderately expert players for items 4, 6, 7 (see Table 6) indicated different goals and plans as they play video games for fun. However, it seems that expert and moderately expert players nonetheless had some common tactics for progressing in the game, making changes as they moved forward, and before they started playing next time.

**Table 6:** Mean scores of three expertise groups for each item of the PMVGS^*^.

***PMVGS Item***	**Expert (*n* = 10)**	**Moderately expert (*n* = 55)**	**Non-expert (*n* = 64)**
	***M***	***M***	***M***
1. I know the physical places where I can play the best.	65.05	27.02	8.20
2. I would stay up all night if it meant a high score in a game.	49.84	21.58	6.91
3. I would rather play a game than spend time with friends and family.	35.34	9.34	4.02
4. I plan the things I have to do next in order to complete the game.	62.53	32.74	11.39
5. I set specific goals for myself (e.g., today I will beat level 60).	41.99	22.99	9.86
6. I play as long as I can.	58.15	23.87	8.87
7. I know which level I will reach before I stop playing.	40.73	19.16	8.73
8. I review my movements to see my progress.	48.47	20.34	7.11
9. I communicate with other gamers to find out how well I am playing.	34.62	19.10	3.65
10. I find myself analyzing the usefulness of strategies while I play.	57.72	30.06	10.45
11. I find myself pausing regularly to check my play.	24.43	16.32	3.76
12. I frequently evaluate my progress.	50.55	23.38	10.26
13. I use game help menus to find solutions to game challenges.	36.79	16.30	9.03
14. I watch other people play and then I do what they do to beat the game.	40.72	28.65	12.05
15. I play a game over and over again to beat the game.	62.09	35.01	12.99
16. I use my own strategies to get through each level.	62.54	35.96	20.45
17. I monitor my playing to pass a challenging level of a game.	25.44	14.18	4.60
18. I play with a partner to progress more quickly.	40.22	21.45	8.44
19. I explore and try different things until I find something to beat the game.	60.42	33.69	13.04
20. I consult with my gamer friends to get help about a game.	42.89	22.58	8.11
21. I check my score to see my progress in a game.	58.68	32.75	15.04
22. I pretend I am one of the best players in this game and play like them.	30.63	12.77	4.41
23. I think of new ways to pass a challenging level of a game.	51.40	25.86	11.21
24. I ask myself if I have considered all options, when playing challenging level of a game.	40.18	26.28	8.66
25. I ask myself if there was an easier way to complete a game after I finish one.	41.72	22.17	8.67
26. I am open to suggestions to improve my play.	75.52	40.20	21.78

* *100 point scale*.

There were no significant differences between moderately expert and-non expert players on items 3, 13, and 22, suggesting that both moderately expert and non-expert players preferred to play a video game instead of spending time with their friends and family, using game help menus, and pretending as if they were the best player in the game and playing like them.

No significant differences appeared between expert and moderately expert players in some items (See items 5, 9, 11, 14, 17, 18, 20, 22, 24, and 25 in Table 6) representing the goal setting, enacting, and adapting phases of self-regulation. As a result, it could be said that expert and moderately expert video gamers did not differ in reporting setting specific goals for themselves as they played video games for fun, communicating with others to assess how they are playing, pausing regularly to check their play, watching others to emulate what they did to win the game, monitoring their play to overcome the challenges, playing with somebody to move forward quickly, talking to their friends to get help, pretending as if they are the best gamers in the game and playing like them, and asking themselves if they considered all the options for winning a game and if there might easier ways to successfully complete the game.

In summary, from the perspective of Winne's model, both moderately expert and non-expert players can be seen to be very different from the experts in the way they set goals, planned their strategies, and adapted their play, and were particularly distinguishable from one another regarding their use of tactics and creating conditions for playing video games, such as time and places to play. In order to understand these results in greater depth, a second, qualitative portion of this study was conducted. Results of this qualitative investigation are presented in the following section.

## Phase 2: qualitative data collection, analysis, and results

### Qualitative participants and data collection

Because of the present study's focus on a specific phenomenon—video game playing—purposeful sampling in which participants are chosen due to a special experience with a phenomenon (Creswell, 2012)—was used for the qualitative portion of the study. Also due to the explanatory nature of this second phase of this study, the researchers focused on typical cases in each expertise group (expert, moderately expert, and non-expert) (Baumann, 1999; Creswell, 2002) in order to provide more representative explanations about self-regulation in video game playing expertise groups. These participants were chosen based on typical responses to survey items 1, 2, 3, 4a, 5a, 5b in the quantitative phase of this research.

Four respondents selected from each group (expert, moderately expert, and non-expert) were contacted via email. As indicated by Creswell and Plano Clark (2007), this number of participants is sufficient for most case studies. A second email was sent as a reminder a week following the original email. One participant from each group chose not to participate; thus a total of nine participants, three from each expertise group, took part in the qualitative phase of this study.

As mentioned previously, the content of the interview protocol was based on quantitative results from the first phase of the study. Since the aim of follow-up qualitative phases in QUAN
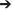
qual designs typically is to explore and elaborate on the results of the statistical tests (Creswell et al., 2003; Creswell and Plano Clark, 2007; Creswell, 2009), the researchers focused on how variables such as amount of video gaming expertise and time devoted to playing video games might contribute to the participants' self-regulation during video gaming for fun. Nine open-ended questions were utilized to explore factors that showed a statistically significant difference related to student self-regulation in recreational video game playing. The interview protocol included questions asking how the participants described themselves as video game players, what they did to better understand rules of a game, how they progressed in a game, and how they tracked their progress.

The interview protocol was pilot tested on two college students and revised based on the results of the pilot testing. Interviews were conducted electronically via Skype and Google Hangout. In order to protect participant privacy, interviews were conducted in a private room using a web camera. Participants were read the confidentiality statement and given the opportunity to terminate the interview at any time. Each participant gave their oral consent and interviews were audio-taped and transcribed. Interviews lasted approximately 10–15 min and at their conclusion participants were thanked and debriefed.

### Qualitative data analysis

Interview transcripts were analyzed using eclectic coding (Saldana, 2009). A key assumption of this approach is that researchers should be open during data collection and reviewing to considering possible options before determining which coding method or methods will yield the most substantive analysis. According to Saldana, coding can be broken into first and second cycles, each with its own methods. Since the primary purpose of our qualitative interviews was to better understand and elaborate on quantitative findings, we used descriptive coding methods in the first cycle and pattern coding in the second.

*Descriptive coding* summarizes basic pieces of the qualitative data in a word or two, with these codes leading to underlying idea of the passage rather than being abbreviations of it. In other words, descriptive coding generates a basic vocabulary from the data to create the elements of each category for analysis. *Pattern coding*, in contrast, helps researchers bring related pieces all together to build more meaningful units. These units can be considered a meta-code, meaning they can be used to group preliminary codes into a smaller number of themes or constructs (Saldana, 2009). In the first cycle, the descriptive coding process was conducted in the margins of the interview transcripts. In the second cycle, similar excerpts from descriptive coding then were put in a same cell in a table to create codes and themes.

In order to provide qualitative validity, methodologists have suggested several procedures, including triangulation, member checking, using an external auditor, and peer debriefing (Creswell, 2009), but have not outlined specific strategies for selecting validation strategies. For the purpose of this study, the researchers used member checking in which a final report or themes are taken back to the participants to determine whether they agreed on the meanings of themes and felt that they were valid. Peer debriefing, in which a person other than the researcher reviews and asks questions about the qualitative study (Creswell, 2009), also was employed for further validation of the study's qualitative findings.

In summary, in order to extend our understanding about self-regulation processes in recreational video gaming and compare experiences of those in three expertise groups, a multiple case study approach (Creswell, 2006) was utilized. First, the researchers read the nine interview transcripts several times in order to code them and extract themes. In the first cycle, ties to Winne's SRL model, general characteristics of experts, expected and unexpected phrases, and passages were highlighted. Based on the codes resulting from use of a descriptive coding method, a table organized by codes and excerpts was created and patterns of association identified. In the second cycle, using a pattern coding method, the researchers brought related codes together and created themes.

### Qualitative results

Five themes emerged from qualitative analysis of the interview data. The first involved participant views about characteristics specific to an expert video gamer, while the remaining four provided information about how video gamers experience the processes of self-regulation in video gaming for fun, including conditions for playing a video game, figuring out a game, how gamers act (before, during and after a game play), and game context. The role(s) and functions of these five themes then were analyzed across all three expertise groups.

The first theme, *characteristics of expert video gamers*, emerged from participant responses about expert video game players themselves. One of these characteristics, identified by every participant, was that they *spent a lot of time* with playing video games and practicing their skills. A second characteristic identified by all interview participants was that an expert video gamer *almost always wins* either at different levels of a game or against an opponent. Video game experts also indicated a third characteristics—that they *had a variety of strategies to play with.* Statements of moderately expert and non-experts illustrate this clearly. Mersin, a moderately expert gamer, shared that an expert is someone who is “…looking up all the different like ways to win game or to better (their) score.” Dolunay, a non-expert, stated “…they talk to their friends about different ways to beat the games.” The fourth and fifth characteristics of expert game players were constructed from statements of expert and non-expert game players. Participants indicated that an expert video game player *understands every game quickly*. Finally, a fifth characteristic of expert game players that both expert and non-expert participants agreed upon was that expert players were *skillful and experienced*. It seemed that expert participants focused more on experience and non-expert participants emphasized becoming more skillful and competitive.

#### Conditions for playing a video game

This theme had mostly to do with where, when, and with what video gamers played video games and how they viewed themselves as video game players. In general, participants in the qualitative part of this study reported playing video games both on and off campus, with expert participants mostly reporting that playing video games in their own home or room and their parents' or friends' homes.

Almost all participants reported playing video games anytime they were available. Moderately expert and non-expert video gamers tended to report that they played on the weekends or school break, while expert video gamers reported playing almost any time they had free time. Koray, an expert participant, stated “I would just say (I play) almost whenever, usually in my free time.” Likewise, Canay from the same group shared “…pretty much whenever I have a free minute I'll do it.” Mersin, from the moderately expert group, said “…when I have free time, like before class (when) I don't have like anything else that I need to be doing. So just more of a free time type of activity.” Dolunay, from the non-expert group also agreed “When I have free time. More on breaks, like summer break and like winter break.”

The devices participants reported playing also varied by level of expertise. Non-expert participants mostly reported playing on their phone, while moderately expert participants indicated playing with their phones as well as on computers and special game devices such as the *Wii*. Tamay, a moderately expert participant, agreed “…most video games that I play are like *Wii* or stuff that (is) on my phone.” While Hanay, from the non-expert group, stated “If I've ever play video game (sic), it is usually on my phone,” Gurelay, an expert participant, indicated using a special game device by saying “I have an *Xbox* which is set up in my living room,” Self-reported confidence of participants also varied by level of expertise, with expert participants being more confident than their moderately expert and non- expert counterparts.

#### Figuring out games

The variety of approaches by which game players began interaction with a game varied along such dimensions as visiting a game's affinity space or discussion group or by observing a group of game players. This theme was clear through participant statements about how they attempted to figure out game rules and context, monitor and control their current play, and utilize prior experiences. Specifically, participants from the expert group—more than the other two groups—reported that they preferred to “…go straight in to the video game and figure it out as I am going” (Canay). Similarly, Koray, another expert, stated that “I am more of a player that (goes) through and mash buttons—like I try to figure it out myself before I go to read the tutorials or something like that.” Expert participants also reported using monitoring and control processes more the other two groups.

As participants were figuring out the game they sought affordances in the environment in order to progress. Two experts and one non-expert participant talked about this in the qualitative interviews. Canay and Gurelay, respectively, stated “…you skim to the menu to see what's available to you” and “I usually look up the control buttons other than that I usually figure that out as I go.” Hanay, from the non-expert group, noted “…just figuring out like the points I guess. So like for *Temple Run* you have to get like X amount of coins in order to like move on to like a harder level.”

#### How gamers act

This theme included participants' statements about their goals and tactics, the way they seek help, and how they adapt play for the next time. In regard to goal setting, expert and moderately expert participants reported setting goals more than the non-expert participants. However, although the expert and moderately expert participants implicitly indicated that they set specific goals, the non-experts explicitly noted forming goals before or during playing video games. Renay, a non-expert, stated “I probably set goals. I guess just kind of for time limits. Say I set goals. Like OK, I can only play until I get 3 stars from this level and then be done.” or “3 stars in these 5 levels and then I'm done.” Similarly Dolunay, a non-expert, shared that “I usually have a goal. I usually try to beat whatever I had last. And if I can't beat the level I keep trying and trying—I don't skip it until I beat it.”

Expert and moderately expert participants reported how they use tactics when game playing while non-experts generally talked about finding the best way to move forward. For instance, Canay, an expert participant, stated:

I like to, extra like (to) just explore the game so if it's like a role playing game or I usually get in that world, then you get to actually explore the role; you can just finish the quest you're supposed to [in order to] go on or you could just run around the world and figure it out. I like to run around the world to figure it out a little bit before actually completing all the quests. I like to complete the game in a 100% entirety so I must figure out what it will take beforehand—what quest that I am gonna have to complete to get that done? Like you can go to, like if you have played or bargained or weapons or anything like that you need to get. You skim to the menu to see what's available to you.

Tamay, a moderately expert participant, reported that “I guess I try to think of what comes next. Because what you do right doesn't really matter so much because you have already kind of thought through it. So you try to make yourself think a step before you are actually doing.”

Hanay, from the non-expert group stated that “I guess (I) just figure out the rules and then I just figure out what best choice to like play” and Dolunay, a non-expert said that “ If something didn't work last time then I don't want to do it again.”

Participants from all expertise groups indicated that their game play changed from game to game. Koray, an expert, shared how he decides how to play, “…it is all very circumstantial. It depends on what is going on in the game.” Gurelay, another expert, reported, “I guess it changes from circumstance to circumstance and from game to game. Different types of the games I assume would be completely different in how you approach them.” Mersin, a moderately expert player, agreed, saying that “…what I really do depends on what game I guess.” Similarly, Renay from the non-expert group stated that “It depends on the game.”

Relative to the help-seeking processes of self-regulation, both expert and non-expert participants reported seeking help. Gurelay, an expert, noted “For some games, like *Skyrim*, I look up some stuff on the internet just to figure out what I am doing.” Canay, another expert, gave this example:

…and if I am stuck in a level and need help, say I can't get past this level, it is going to drive me insane. What can I do? So they usually are more than willing to help me as I am figuring (something) out or if it's a friend I call them.

But non-experts also reported seeking help. One of these, Renay, stated that “…looking at online strategies that other people have used and maybe even finding cheat codes.” Dolunay, another non-expert, indicated that “If I don't understand the game I'll ask maybe a friend who plays the same one.”

#### Game context

The game context theme refers to how the video game players used the game environment to support them in monitoring their progress in the game, whether they talked about a game with anybody, and whether the way they studied was similar to the way they played video games. The majority of moderately expert and non-expert participants reported relying on the game itself to keep track of their progress while they are playing. One non-expert participant, Hanay, when asked how she tracks her progress stated “…usually it [the game] keeps track.” Tamay, a moderately expert player, noted that “During the play I guess usually the computer does it for me. But you can also keep tracking in your head like how many coins that I can get on the last one. Like if count above like how many coins that you have over the top you can keep counting as you go through.” Moderately expert and non-expert participants also reported talking with others about a game.

Unlike moderately expert and non-expert participants, however, all of the expert participants made associations between how they studied and how they played video games. Canay, an expert, stated: “Basically, I would say like the way I map out my homework to get my homework done and making sure I am completing everything would be the same as the way I would map out a game to see that I am actually completing everything in that level to pass forward to it. I kind of try to make sure I cover the basics and I (have) done everything that I need to be done. So that's very similar and I haven't thought it before but that's very similar. Koray stated that “…because of like I said if I know something is not working then I am not gonna do it again. I guess that kind of trial and error aspect is similar.” Along the same line Gurelay added, “Actually, I‘ve learned a lot of history from playing certain games. It helped me with umm I guess some of my history classes I suppose.”

## Discussion

Both quantitative and qualitative analyses comparing video game players in the three expertise groups revealed significant differences in self-regulation favoring the experts. Specifically, expert game players used processes of self-regulation considerably more than moderately expert players, and moderately expert participants self-regulated more than non-experts (see Table 7). These findings are consistent with the majority of studies in the self-regulation and expertise literature more generally (e.g., Lefebvre-Pinard and Pinard, 1985; Glaser, 1987; Zimmerman and Risemberg, 1997; Zimmerman, 1998, 2002b; Cleary and Zimmerman, 2001). For instance, Cleary and Zimmerman (2001) worked with basketball experts and non-experts in order to determine possible differences in their self-regulatory processes regarding free-throw shooting. Their results indicated that the experts self-regulated more than the non-experts.

**Table 7:** **Summary of quantitative and qualitative findings**.

**Self-regulation phases**	**Expert**	**Moderately expert**	**Non-expert**
Definition of task	Play video game At special places such as their room or friends house Any time Mostly with special devices such as game consoles and computers	Play video game On and off campus At weekend and school break Mostly with their phones and computers	Play video game On and off campus At weekend and school break Mostly with their phones and computers
	They have lots of game experience in wide variety of video games		
Goal setting and planning	Implicitly stated their playing goal	Implicitly stated their playing goal	Explicitly stated their playing goal
	Their goals were mostly game specific goals	Their goals were mostly game specific goals	Their goals were general game playing goals such as limiting time to play
	They are more committed to play game than other two groups		
	They plan things ahead of time then take action		
Enacting tactics	Purposefully select the tactics among their large tactics repository	Use less monitoring tactics than the experts	Use less monitoring tactics than the moderately experts
	Use more monitoring tactics according to their play	Use tactics mostly include other players	Monitor based on the games' feedback
	Use all available tactics		
	They are more willing to get help from social environments		
Adapting meta-cognition	They ask themselves if they have considered all options, when playing challenging level of a game.	They ask themselves if they have considered all options, when playing challenging level of a game.	They generally don't think of their plays
	They ask themselves if there was an easier way to complete a game after they finish one.	They ask themselves if there was an easier way to complete a game after they finish one.	
	They think of new ways to pass a challenging level of a game.		
	They open to suggestions to improve their play.		

Following a similar approach, the present researchers followed up the *PMVGS* scores of three expertise groups with *post hoc* comparisons for individual *PMVGS* items. The researchers grouped and discussed the items according to each phase in Winne's SRL model to better identify differences and similarities among three video game expertise groups regarding use of self-regulation in playing video game for entertainment. Winne's (2001) first phase, *definition of task*, indicates a process in which individuals identify the external and internal conditions needed to perform a task. In results corresponding to Winne's first phase, expert and non-expert participants of this study significantly differed from each other regarding where and when to play video games. The expert game players reported the physical places where they could play their games most successfully more specifically than the non-expert participants. Also, moderately expert players more clearly identified the locations for their play than did the non-experts. This could imply that the experts in video game playing tend to be more cognizant of their surroundings and the effects of their environment on their game play, much like experts in other domains (Zimmerman, 2006).

These quantitative findings were supported by our qualitative results, in which expert participants described playing mostly at their homes or rooms, which may indicate they needed or preferred a mostly undistracted place. For example, Koray from the expert group noted, “I play at my house very often—otherwise at (a) friend's house.” As indicated in Winne's model of self-regulation, conditions are important inputs to the self-regulated learning process. Furthermore, prior SRL studies have shown that the more students were aware of task requirements, context, and their surroundings, the more likely they are to use processes of SRL and succeed in a task (e.g., Jamieson-Noel, 2005; Schellings and Broekkamp, 2011; Greene et al., 2012). Similarly, the findings of the current study show that a choice of where and when to play games also is part of the self-regulation of video game playing. Similar to highly self-regulated learners in general (see Winne, 2005), video game players seem to benefit from being aware of contextual factors, in their case features of video games and the physical environments in which they are playing.

Since game play experiences are products of unique interactions between a game and a game player's sensations, thoughts, feelings, actions, desires, anticipations, prior game play experiences and tips or tricks that they heard from others, they impact both the success and satisfaction of game players (Ermi and Mäyrä, 2005). It may be, however, that researchers and practitioners from the field of education can use these findings to better understand how experts' knowledge and experiences about evaluating games, contexts, and self for video game playing might transfer to more formal learning settings.

In the present study, expert game players differed significantly from the moderately expert and non-expert players in how they responded to items representing Winne's goal setting and planning phase. This result was expected based on findings of previous studies in the expertise and self-regulation literature (e.g., Kitsantas and Zimmerman, 2002; Cleary and Zimmerman, 2004), which suggest that a player's actions and reactions in the game likely will create a dynamic relation between a player and a game. Based on players' goals, their active and conscious inferences, and their decisions to modify and interpret current playing goals, this dynamic relationship is likely to be sustained (Zaparyniuk, 2006). Also, just as with highly self-regulated learners in general (e.g., Cleary and Zimmerman, 2004), responses of expert and moderately expert participants did not significantly differ in their responses to an item signaling a general goal (Item 12, “I set specific goals for myself.”). However, the expert participants rated items indicating their commitment to game playing (e.g., Item 6, “I play as long as I can.”) significantly higher than moderately expert and non-expert participants.

These quantitative findings with respect to goal setting and planning were complemented by the qualitative ones. Expert and moderately expert participants, for instance, reported setting more game specific goals than non-experts, although they did not explicitly state how they formed these goals. Canay, one of the experts, shared “…in role playing games I like to explore *where I am actually at* versus just doing the tasks required to complete the game.” Tamay, from the moderately expert group, similarly stated that “I want to get not to just the next level but I want to get as many coins as I can get.” Dolunay, another moderately expert participant, indicated that “I usually have a goal. I usually try to beat whatever I had last.” On the other hand, non-expert participants tended to indicate setting general game playing goals such playing for a certain amount of time or until passing a certain level.

One particularly interesting finding regarding the *goal setting and planning phase* was that the goal setting of expert and moderately expert participants for video game play seems to have been more implicitly than explicitly recognized by these players. For instance, when the researchers directly asked players if they had set specific goals their initial answers were mostly “No.” As they described features of their play, however, several of them realized that they in fact had set goals and by the end of the interview were explicitly reporting having set goals. One reason for this could be that they may not have considered video game playing as a serious activity during which they might form goals in the same way they might have for learning academic subjects or performing other tasks. Another reason might be that, because they had a high enough expertise level to have automatized this part of game playing, they did not realize they in fact were setting goals. Descriptions of goal setting provided by non-expert participants seem to provide further support for this claim. Although, these non-expert players stated that they set goals, theirs were mostly aligned with the goals pursued by all video game players generally, such as passing a level. In contrast, expert participants' goal statements were highly game-specific and included their plans to reach the goal even though they did not directly report setting goals for their play. Thus, it may be useful for educators to guide students in the goal setting and planning components of self-regulation to help them recognize connections between setting short-term goals and mastery of complex skills and content.

The other characteristic of expert participants was deploying strategies for play quite purposefully. By using their existing knowledge of tasks, expert and highly self-regulated learners generally can strategically select, control, and monitor strategies to attain their goals (Ertmer and Newby, 1996). Less self-regulated learners, however, typically need more explicit direction in applying strategies to learn effectively (Winne, 2005). As in earlier studies of self-regulation (e.g., Zimmerman, 1990; Weinstein and van Mater Stone, 1993; Ertmer and Newby, 1996), the present study's expert game playing college students significantly differed from their non-expert counterparts in their responses to all of the items representing the *enacting tactics phase*, which included *monitoring, help seeking, and use of play related tactics* in Winne's (2001) model (Items 8 through 22). An interesting finding here was that on the items indirectly inquiring about use of monitoring processes (Items 8, 10, 12, and 21), each of the three groups significantly differed from the others. That is, expert participants, when compared to the moderately expert and non-expert participants, reported using more *monitoring* processes of self-regulation in their game play, as did the moderately expert participants as compared to the non-expert participants.

Both expert and non-expert participants in the present study quite clearly indicated that they *monitored the success of the strategies* they used in their game playing, but seemed to differ greatly in what they monitored. For instance, Gurelay, an expert participant, stated that he carefully monitored how effective his strategies were, so that “…if a match went very badly”…he usually switched strategies and “…if the strategy's consistently bad then I definitely switch.” In contrast, non-expert participants primarily relied on their game progress to monitor their play, focusing more on game outcomes (e.g., number of points) rather than on the strategies that produced those outcomes.

This difference in focus perhaps can be explained by the dissimilarities within the goal setting and planning phase where the expert participants set more game-related goals than moderately expert and non-expert participants. Presumably, the expert players could be more specific and purposeful about what they were aiming for and the steps they believed were needed to accomplish their goals (Butler and Winne, 1995; Butler, 1997; Zimmerman and Kitsantas, 1997). Moreover, application of self-regulation involves not only awareness and utilization of available strategies but also evaluation of the strategies' appropriateness and usefulness. Game-playing experts in this study, similar to experts in other domains (see, for example, Isaacson and Fujita, 2006), demonstrated that they have a repertoire of playing strategies and know what they mastered and what they have not. This knowledge, of course, has a direct impact on every dimension of self-regulation.

According to participants' accounts of their game playing as gathered in the qualitative phase of the study, each game or game genre has specific features such as points, time restrictions, levels etc. that influence the way players set goals, choose tactics, and monitor their progress. For example, in an electronic tennis game such as in *Wii Sports*, players can follow tennis rules and set their goals as in a real tennis match. Holding a real racket is different than swinging the motion sensor as a racket, however. Depending on their experience level in actually playing tennis, electronic game players could have varying goals—from holding the motion sensor right to winning at least one set—and might use various monitoring tactics. Thus in order to shed more light on the association between use of goal setting and monitoring progress in playing video games, researchers need to focus in on specific game or game genres, players' prior experience in this domain, and using observations and think-aloud methods as players play. With further studies of interactions among goal setting, use of strategies and self-monitoring, video game playing learners could gain skill at using self-monitoring processes in video games. Then, with the help of their instructors and co-learners they presumably could transfer this skill to a variety of learning environments.

Individuals in the expert group also differed from the non-expert groups in the extent to which they reported using *play-related tactics* (Items 9, 14, 15, 16, 18, 19, and 22). Some studies in the literature (e.g., Lindner and Harris, 1992; Weinstein and van Mater Stone, 1993; Ertmer and Newby, 1996) have indicated that in order to self-regulate their learning and be successful, expert learners deploy several types of knowledge, including knowing how to use cognitive (e.g., memorizing), motivational (e.g., recalling earlier successes), and environmental (e.g., working with a group) strategies. Like expert learners generally, the expert video game players in this study reported applying a variety of tactics in their game play, including using them to progress in the game (cognitive), pretending they are one of the best players of the game and playing like them (motivational), and consulting with game-playing friends to get help (environmental).

Although, the expert and non-expert participant responses differed significantly in all items representing the enacting tactics phase of self-regulation, for some play-related tactic items (Items 9, 14, and 18; e.g., Item 14 “I watch other people play and then I do what they do to beat the game.”) this difference did not appear in comparisons of expert and moderately expert participants. On close examination, it was noted that members of both groups included other gamers' inputs to their present game play. That is, in order to play better both expert and moderately expert participants reported utilizing tactics tied to other video game players, including judging how they are playing (Item 9), emulating successful players (Item 14), and co-playing with another player (Item 18). In other items related to tactics that included just themselves and no other players (e.g., Item 16, “I use my own strategies to get through each level.”), the expert participants scored significantly higher than moderately expert ones.

In contrast to the expert players who tended to apply all available tactics—their own and others—moderately expert participants mostly relied on tactics that included other players. This could mean that the experts possess a larger repertoire of tactics than moderately expert and non-expert participants, consistent with earlier studies of expertise (e.g., Chi, 2006). One possible explanation for this is that in becoming a more experienced video game player, the moderately expert players benefit from others' game play. They may take others as models to learn from their play to achive their goals (Zap and Code, 2009). Therefore if video gamers play with more expert partners they can succeed by reducing time and effort ordinarily taken up by trial and error. As in SRL more generally (Hadwin et al., 2005), if a gamer observes and imitates other gamers –especially experts—they will realize that to win they need to use their own tactics, while taking advantage of available social and environmental support. The expert participants also provided more details about the tactics they used than the moderately expert and non-expert participants. Koray, an expert, stated that “it is all very circumstantial. It depends on what is going on in the game.” Similarly, Gurelay pointed out, “I guess it changes from circumstance to circumstance and from game to game. Different types of the games I assume would be completely different in how you approach them.” Future studies thus might profitably focus on how expert video game players use tactics in a specific game compared to moderately expert or non-expert players. Among possible areas of focus might be examining how game players decide to switch one tactic for another and whether there are be any differences or similarities among expert and less expert participants regarding changing tactics.

Unlike other dimensions in the *enacting tactics* phase in which expert and moderately expert participants did not differ, expert participants indicated being more willing than moderately expert participants to get suggestions from social environments to play better. One of the experts, Canay, stated: “If you are playing a game, it is not always yourself. There is always somebody else playing with you or even if they're not playing with you at that moment they are still there to help you.” Future studies could examine how social environments might impact experts' and others use of self-regulation processes in video game playing. Moreover, participants' help seeking from such internet sources as games' web sites and affinity spaces (where players share tips and tricks on passing a level or completing a task) could be analyzed in terms of what self-regulation processes are driving these visits.

Finally, among the items addressing the *adapting metacognition* phase (Items 23–26, e.g., item 26 “I ask myself if there was an easier way to complete a game after I finish one”), expert game players reported more adaptations in their play from one time to the next than did the non-experts. Specifically, expert players on average rated items focused on their thinking of new ways to pass a challenging level of a game and being open to suggestions to improve their play higher than the moderately expert players. For instance, Koray, from the expert group, shared the following view: “You can see how I play right after the game and what kind of progress there is. In sports games they put the stats right in front of you so you don't have to think about whether or not you did well, all you have to do is look. But for *Call of Duty*, if things aren't going well, I will do a kind of mental inventory of the things that I have tried and what is and isn't working.”

## Conclusion

This research shows that expert video game players use many more processes of self-regulation than less expert video game players. For every one of the 26 items on the *PMVGS* and across all the categories of self-regulation represented (Winne, 2001), expert video game players reported using significantly more self-regulation than non-experts. Similarly, moderately expert video game players rated their use of self-regulation processes as greater than non-experts in all but three of the PMVG items. In the second phase of the study, qualitative findings were highly consistent with the quantitative results.

Although this study clearly demonstrates that expert video game players use more self-regulation processes than moderately expert and non-expert video game players in playing video games, replication is needed with larger samples of players and with players interacting with games in a variety of settings (e.g., with particular games or game types). Also, given that the quantitative portion of the current study was conducted using a convenience sampling method, future studies aimed at understanding how the self-regulation of expert and less-expert game players in varying age groups might differ should be valuable. Focusing especially on specific dimensions of self-regulation utilized by expert players (e.g., their goal setting, use of tactics,) likewise should lead to better understanding of how game-related self-regulation processes might be generalized to more academic tasks and contexts. That is, understanding how expert video game players manage these processes may help educators encourage student learning and cognitive growth by exploiting commonalities between successful game playing and self-regulated learning more generally (Gee, 2003).

Finally, it should be noted that the current study—as a preliminary exploration of self-regulation processes in recreational video game playing—did not focus extensively on particular video games or game types. However, both participants' statements and the researchers' observations clearly indicate that different types of games require varied skills and modes of self-regulation for mastery. For example, in a first person-shooter game (e.g., *Call of Duty*) players must become familiar with their weapons and practice using them, as well as improving their map reading skills. In a strategy game (e.g., *Age of Empire*), in contrast, players need to determine their goals before taking next steps and to analyze neighbors' investments in order to allocate their resources successfully. In light of such differences, future studies examining interactions between specific game features and the application of self-regulation processes almost certainly will prove to be valuable.

## Authors note

The present study is based on the first author's PhD dissertation at the University of Nebraska, Lincoln, entitled: Exploring Self-Regulation Of More And Less Expert College-Age Video Game Players: A Sequential Explanatory Design.

## Author contributions

Substantial contributions to the conception or design of the work; or the acquisition, analysis, or interpretation of data for the work (MYS, RHB). Drafting the work or revising it critically for important intellectual content (MYS, RHB). Final approval of the version to be published (MYS, RHB). Agreement to be accountable for all aspects of the work in ensuring that questions related to the accuracy or integrity of any part of the work are appropriately investigated and resolved (MYS, RHB).

### Conflict of interest statement

The authors declare that the research was conducted in the absence of any commercial or financial relationships that could be construed as a potential conflict of interest.
